# Prevalence of congenital heart disease among school children in Qinghai Province

**DOI:** 10.1186/s12887-022-03364-5

**Published:** 2022-06-08

**Authors:** Shangfei He, Fengqing Zhao, Xudong Liu, Fangzhou Liu, Yumei Xue, Hongtao Liao, Xianzhang Zhan, Weidong Lin, Murui Zheng, Junrong Jiang, Huoxing Li, Xiaofeng Ma, Shulin Wu, Hai Deng

**Affiliations:** 1 Qinghai Province Cardio Cerebrovascular Disease Specialist Hospital, No.7 of Zhuanchang Road, Xining City, 810012 Qinghai Province China; 2 Department of Cardiology, Guangdong Cardiovascular Institute, Guangdong General Hospital, Guangdong Academy of Medical Science, No.106 of Zhongshan Second Road, Guangzhou City, 510080 Guangdong Province China; 3 School of Public Health, Sun Yat-Sen University, No.74 of Zhongshan Second Road, Guangzhou City, 510080 Guangdong Province China; 4 Guangzhou Center for Disease Control and Prevention. , No.23 of Jiaochang Road, Guangzhou City, 510120 Guangdong Province China; 5 Southern Medical University, No.1023-1063 of Shatai South Road, Guangzhou City, 510515 Guangdong Province China

**Keywords:** Prevalence, Congenital heart disease, School-aged children, Qinghai plateau

## Abstract

**Objectives:**

This study aimed to investigate the prevalence of congenital heart disease (CHD) among school children in Qinghai province, a high-altitude region in China.

**Methods:**

A cross-sectional study was conducted among school-aged children in 2019. All subjects completed a survey with a structure questionnaire and underwent CHD screening. CHD was screened by standard physical examination and further confirmed by echocardiography. Multivariate logistic regression were used to estimate the association of CHD prevalence with gender, nationality, and altitude.

**Results:**

A total of 43,562 children aged 3–19 years participated in the study. The mean (SD) age was 11.2 (3.3) years. 49.7% were boys, and 80.0% were of Tibetan. CHD was identified in 293 children, with an overall prevalence of 6.73 ‰. Among them, 239 were unrecognized CHD, yielding a prevalence of 5.49 ‰. Atrial septal defect accounted for 51.9% of the CHD, followed by patent ductus arteriosus (31.1%), ventricular septal defect (9.9%). The CHD prevalence was significantly higher in female (8 ‰), Han race (18 ‰), children lived in Qumalai county (13 ‰), and children lived in a higher altitude (13 ‰). Female had greater prevalence of total CHD, atrial septal defect, and patent ductus arteriosus, but insignificant difference was observed in ventricular septal defect prvalence than male. In multivariable logistic regression analyses, female (OR, 1.48; 95% CI, 1.17–1.87, *P* = 0.001), Han population (OR, 3.28; 95% CI, 1.67–6.42, *P* = 0.001), and higher altitudes (OR, 2.28; 95% CI, 1.74–3.00, *P* < 0.001) were shown to be independently association with CHD prevalence.

**Conclusions:**

The prevalence of CHD in Qinghai province was 6.73 ‰. Altitude elevation, female, and Han population were independently association with CHD prevalence.

## Introduction

Congenital heart disease (CHD) is cardiovascular malformation in young children, caused by abnormal cardiovascular development in the fetus [1]. CHD is strongly associated with heart dysfunction and death [2]. Interactions of genetic with environmental elements have been reported as pathogenic factors associated with CHD [3]. Previous studies showed that the prevalence of CHD varied largely in different races [4]; additionally, epidemiological study has found that the high altitude with a lower atmospheric oxygen tension was associated with a high prevalence of CHD [5].

The Qinghai-Tibetan Plateau (QTP) is the highest plateau in the world and characterized with geographic and ethnic diversity. There were variation in CHD prevalence among school children lived in Tibet due to the difference of selected survey areas [6–9]. However, CHD prevalence was reported in previous studies were not totally representative of that in the entire territory of Tibet, because of unknown regional differences in this vast land with diverse climates and topographies. Two studies conducted more than ten years ago and found that the total prevalence of CHD in Children in Qinghai province was about 7.2 ‰ [6, 10]; however, the socioeconomic status of Qinghai Province and the health condition of local residents have been significantly improved in the past decades. Nevertheless, it is not clear whether the prevalence of CHD in children in Qinghai province has changed in recent year.

Therefore, we conducted this cross-sectional study to investigate the prevalence of the CHD in children in Qinghai province and to explore the possible influential factors of CHD.

## Methods

### Study population

This cross-sectional study was conducted among school children aged 3–19 years in Qinghai province from January to December, 2019. A stratified multistage random sampling method was adopted to select potential subjects. First of all, two autonomous prefectures (Yushu Tibetan Autonomous Prefecture and Huangnan Tibetan Autonomous Prefecture) were randomly selected in Qinghai Province; secondly, four counties (Jianzha, Tongren, Henan, Zeku) from Huangnan Tibetan Autonomous Prefecture and Qumalai from Yushu Tibetan Autonomous Prefecture were randomly selected; thirdly, all schools and kindergartens in the five selected counties were selected; lastly, all students aged 3–19 years were investigated. Finally, a total of 43,562 school children were included and successfully accomplished the survey. This study was approved by the Ethics Committee of the Qinghai Province Cardio Cerebrovascular Disease Specialist Hospital (Institutional Review Board No. QYLL20190731). This study was conducted in line to the requirement of Declaration of Helsinki and the informed consent was obtained from legal guardian or the parents of each included child. If children were diagnosed with CHD, they were notified by phone to present themselves to Qinghai Province Cardio Cerebrovascular Disease Specialist Hospital for further treatment.

### Data collection

A structure questionnaire was used to collect social demographic information, including date of birth, age, gender, and ethnic group. The altitude of each school was measured (44ST, BARIGO, Starnberg, Germany) according to the standard protocol. Then the altitude was transformed to dichotomous categorical variable: lower altitude (≤ 4200 m) and higher altitude (> 4200 m) [9].

CHD was ascertained according to the following examination. First, each subject underwent a standard examination of cardiac auscultation and peripheral artery palpitation by an experienced cardiologist. Special attention was paid to growth condition and cyanosis. Children who may have abnormal signs of cardiac defect, such as growth retardation, cardiac murmurs, fixed splitting of the second heart sound, and clinical cyanosis. Secondly, subjects with pathologic cardiac souffle or cyanosis were further examined by echocardiography by using a Vivid I cardiovascular ultrasound system (S5, GE Healthcare, Horten, Norway) according to the standard examination protocol [8]. All echocardiograms thought to be abnormal were further reviewed by another expert in pediatric senior echocardiography with more than 5 years work experience to ensure quality. About 5% of children were randomly selected to repeat the examination and two examinations from each child were all consistent. Unrecognized CHD was defined as patients with CHD diagnosed for the first time during the echocardiographic screening. The classification of CHD was based on the International Classification of Diseases, Ninth Revision, and the Clinical Modification code. Ventricular septal defect (VSD) is defined as a left to right shunt at the ventricular level. The blood flow channel between the aorta and the pulmonary artery does not close naturally after birth, and it is defined as patent ductus arteriosus (PDA). Complex CHD includes severe pulmonary valvular stenosis with significant subvalvular obstruction and a right-to-left interatrial shunt, tetralogy of Fallot, transposition of the great arteries with concordant atrioventricular connections and discordant ventriculo-arterial connections, and hypoplastic left heart syndrome. We focus on these severe, commonly occurring lesions because they are frequently used as benchmarks for surgeon and programmatic performance [11, 12]. Patent foramen oval and atrial septal defect (ASD) (defect < 5 mm in diameter) were excluded from CHD.

### Statistical analysis

The prevalence of CHD was calculated and the distribution difference was examined by using Chi-square test or Fisher’s exact test. Univariate and multivariate logistic regression were used to calculated prevalence odds ratio (POR) and 95% confidence interval (CI), to display the association of CHD risk with potential influential factors. Statistical tests were performed using SPSS (version 20.0; SPSS, Inc., Chicago, IL, USA). A two-sided *p* value < 0.05 was considered to be statistically significant.

## Results

### Prevalence of CHD

A total of 43,562 school children (21,664 male and 21,898 female) were screened, The mean (SD) age was 11.2 (3.3) years. 49.7% were boys, and 80.0% were of Tibetan. CHD was identified in 293 children, with an overall prevalence of 6.73 ‰. Among them, 239 were unrecognized CHD, yielding a prevalence of 5.49 ‰. The three most prevalent CHD were ASD (3.5 ‰), PDA (2.1 ‰), and VSD (0.7 ‰). As shown in Table 1, the prevalence of CHD was significantly higher in female than in male (8 ‰ vs 5 ‰, *P* = 0.001). The CHD prevalence differed by counties (*P* < 0.001) and varied in diverse ethnic groups (*P* < 0.05). The CHD prevalence was highest in Qumalai (13 ‰), and followed by Zeku (9 ‰), Henan (7 ‰), Jianzha (5 ‰) and Tongren (3 ‰). The prevalence of CHD was highest in Han (18 ‰), and followed by Tibetan (7 ‰), Mongolian (7 ‰), Hui (7 ‰), and Tujia (2 ‰) population. The CHD prevalence was about 30 ‰, 6 ‰, and 6 ‰ in children aged 6 years or lower, aged 6–12 years, and aged more than 12 years, respectively.

**Table 1:** Prevalence of CHD in schoolchildren in high altitude region

	Total	Non-CHDN (‰)	CHDN (‰)	*P* value
All	43,562	43,269 (993)	293 (7)	
Gender				0.001
Male	21,664	21,546 (995)	118 (5)	
Female	21,898	21,723 (992)	175 (8)	
Race				0.029
Tibetan	35,064	34,830 (993)	234 (7)	
Mongol	5070	5034 (993)	36 (7)	
Han	495	486 (982)	9 (18)	
Hui	1830	1818 (993)	12 (7)	
Sala	212	212 (100)	0 (0)	
Tujia	828	826 (998)	2 (2)	
Others	63	63 (100)	0 (0)	
County				< 0.001
Jianzha (1990 m)	8457	8418 (995)	39 (5)	
Tongren (2480 m)	12,009	11,978 (997)	31 (3)	
Henan (3510 m)	5307	5268 (993)	39 (7)	
Zeku (3655 m)	12,456	12,341 (991)	115 (9)	
Qumalai (4223 m)	5333	5264 (987)	69 (13)	
Age at diagnosis of CHD				< 0.001
≤ 6 years	1740	1687 (970)	53 (30)	
6–12 years	26,510	26,364 (994)	146 (6)	
> 12 years	15,312	15,218 (994)	94 (6)	
Altitude				< 0.001
≤ 4200 m	38,229	38,005 (994)	224 (6)	
> 4200 m	5333	5264 (987)	69 (13)	

*CHD* Congenital heart disease

**Fig. 1:**
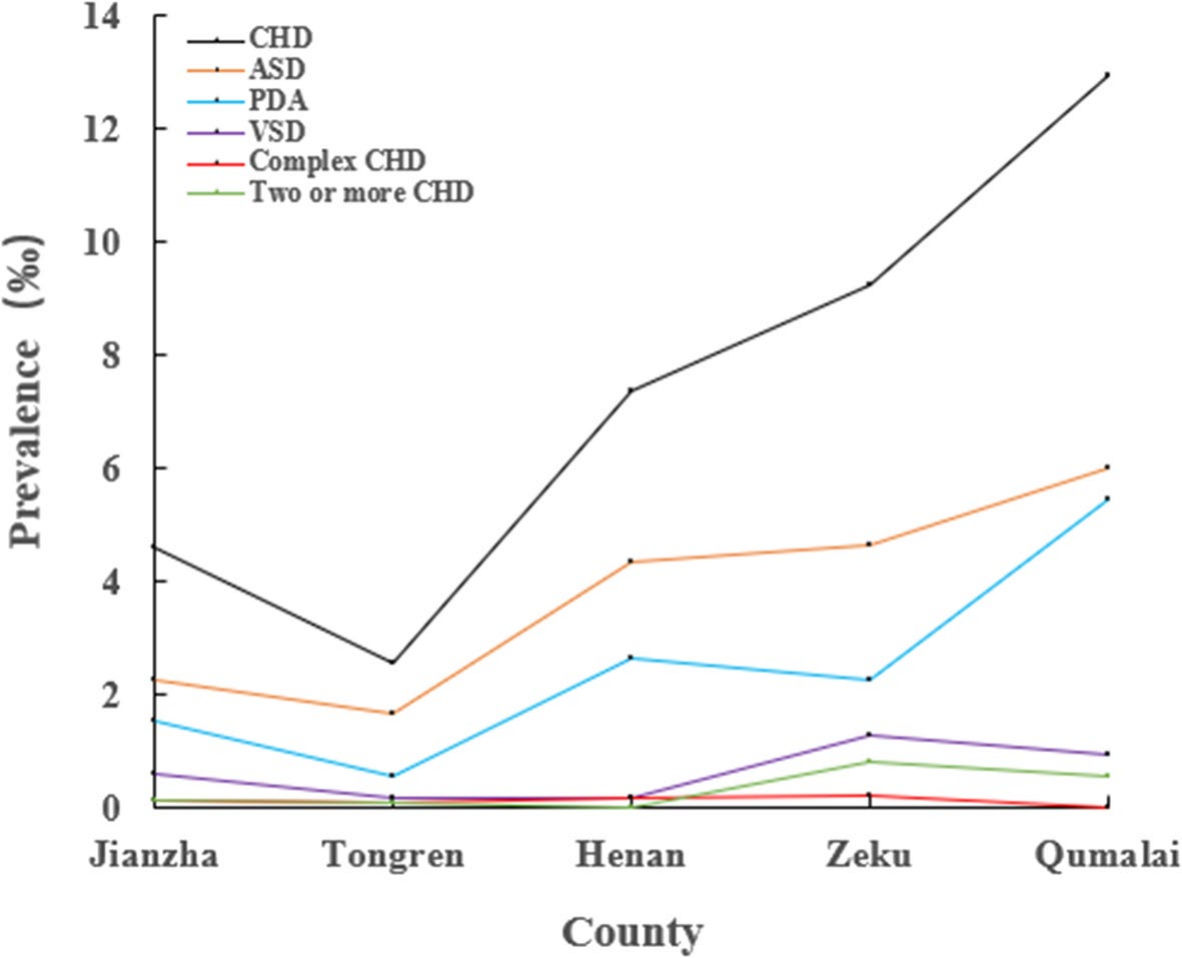
Prevalence of total CHD and its subtypes in 5 selected counties. With ascending altitudes, the prevalence of total CHD, ASD, and PDA increased accordingly. In contrast, the prevalence of VSD, complex CHD, and two or more CHD decreased with increasing altitude. The most common CHD was ASD, while complex CHD were rare in five counties. Black line represents CHD, orange line represents ASD, blue line represents PDA, purple line represents VSD, red line represents complex CHD, and green line represents two or more CHD. Tongren is at an altitude of 2480 m; Qumalai is an area of pastureland at 4223 m. Zeku is at an altitude of 3655 m; Jianzha is at an altitude of 1990 m; Henan is at an altitude of 3510 m

**Fig. 2:**
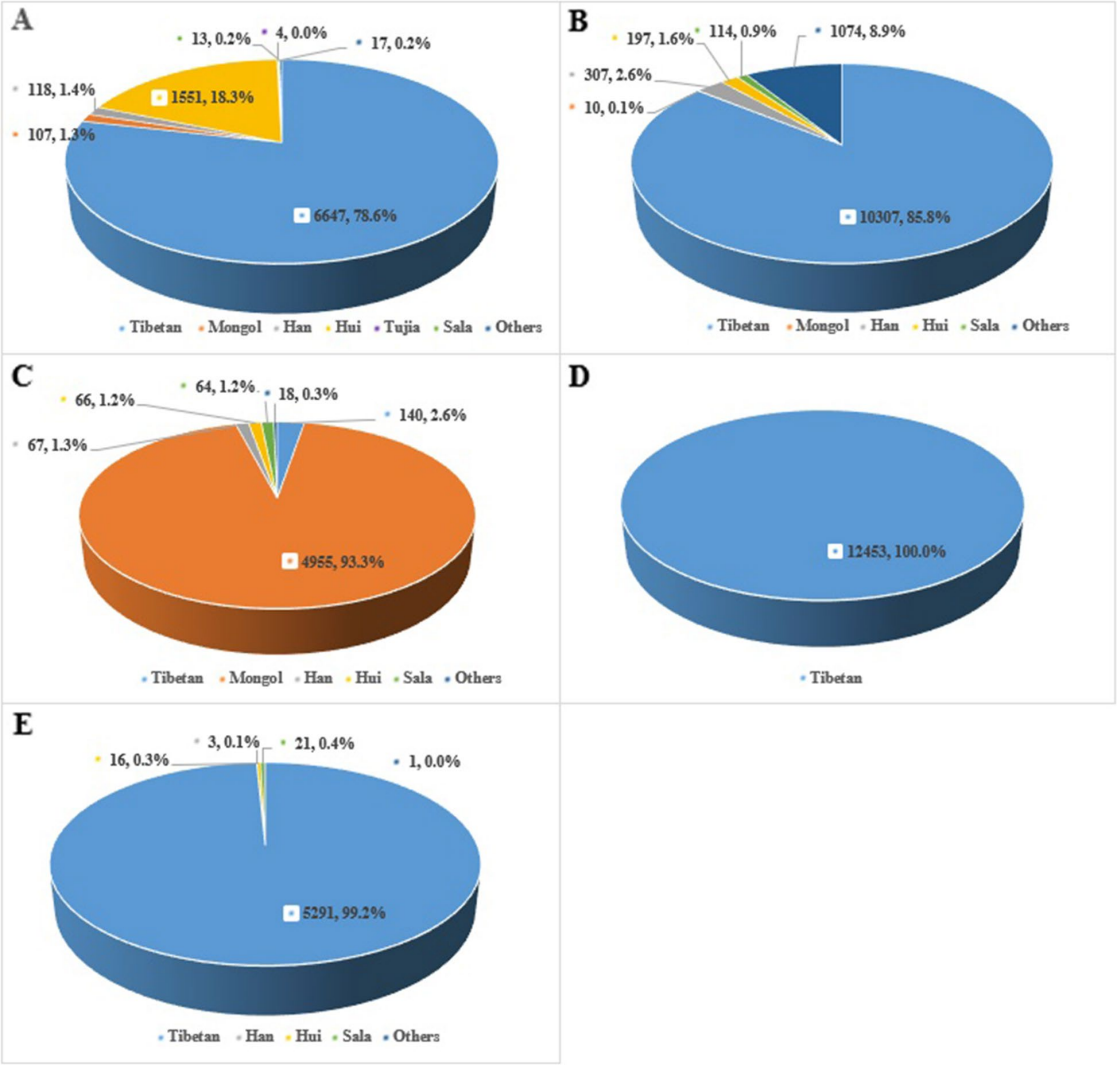
Percentage of each race make up the population in 5 selected counties. The pie chart shows the percentage of each race make up the population of each area. **A** shows the percentage of each race make up the population of Jianzha; **B** shows the percentage of each race make up the population of Tongren; **C** shows the percentage of each race make up the population of Henan; **D** shows the percentage of each race make up the population of Zeku; **E** shows the percentage of each race make up the population of Qumalai. Blue indicates the number and proportion of Tibetan. Orange indicates the number and proportion of Mongol. Grey indicates the number and proportion of Han. Golden indicates the number and proportion of Hui. Purple indicates the number and proportion of Tujia. Green indicates the number and proportion of Sala. Dark green indicates the number and proportion of others

The altitudes of five selected counties are different; Qumalai has the highest altitude of 4223 m and Jianzha is at the lowest altitude of 1990 m. The CHD prevalence was significant higher in children lived in higher altitude than in lower altitude (≤ 4200 m vs > 4200 m: 13 ‰ vs 6 ‰, *P* < 0.001). With the raise of altitudes, the prevalence of total CHD, ASD, and PDA increased accordingly (Fig. 1). The most common CHD in each county was ASD, while complex CHD and two or more CHD were rare. As shown in Fig**. **2, the pie chart shows the percentage of each race make up the population of each area. The number and proportion of Tibetan account for the vast majority in the five survey areas, in addition to the Henan county. With ascending altitudes, the proportion of Tibetan increases, approximately 100% when altitude above 3600 m; the proportion of Hui nationality is the opposite. The Han nationality is mainly concentrated altitude below 3600 m. In Henan County, Mongols account for the vast majority, accounting for 93.3%, followed by Tibetans.

### Gender-specific prevalence of CHD

As shown in Table 2, compared with male, female had greater prevalence of ASD, PDA, complex CHD, and two or more CHD (ASD, 4 ‰ vs 3 ‰; PDA, 3 ‰ vs 1 ‰; complex CHD, 0.2 ‰ vs 0.1 ‰; two or more CHD, 0.5 ‰ vs 0.2 ‰). In lower altitude (≤ 4200 m) group, female had slightly greater prevalence of total CHD, PDA, and ASD than male (CHD, 7 ‰ vs 5 ‰; PDA, 2 ‰ vs 1 ‰; ASD, 4 ‰ vs 3 ‰). The prevalence was greater in female (15 ‰) than in male (11 ‰) in higher altitude. The highest prevalence of CHD in both male and female were in Qumalai and in Han population.

**Table 2:** Gender-specific prevalence of CHD in school-aged children in a high altitude region

	Male			Female		*P* value
	N	(‰)		N	(‰)	
Nationality						0.003
Tibetan	98	(6)		136	(8)	
Mongol	12	(5)		24	(9)	
Han	3	(11)		6	(27)	
Hui	4	(4)		8	(9)	
Sala	0	(0)		0	(0)	
Tujia	1	(2)		1	(2)	
Others	0	(0)		0	(0)	
County						> 0.05
Jianzha (1990 m)	20	(5)		19	(4)	
Tongren (2480 m)	14	(2)		17	(3)	
Henan (3510 m)	12	(5)		27	(10)	
Zeku (3655 m)	43	(7)		72	(11)	
Qumalai (4223 m)	29	(11)		40	(15)	
CHD subtype						0.002
ASD	67	(3)		85	(4)	
VSD	15	(1)		14	(1)	
PDA	29	(1)		62	(3)	
Complicated CHD	2	(0.1)		4	(0.2)	
Two or more CHD	5	(0.2)		10	(0.5)	
Age at diagnosis of CHD						< 0.001
≤ 6	26	(28)		27	(33)	
6–12	51	(4)		95	(7)	
> 12	41	(6)		53	(7)	
Altitude						> 0.05
≤ 4200 m	89	(5)		135	(7)	
> 4200 m	29	(11)		40	(15)	

*ASD* Atrial septal defect, *VSD* Ventricular septal defect, *PDA* Patent ductus arteriosus, *CHD* Congenital heart disease

### Association of CHD risk with influential factors

As shown in Table 3, based on multivariable logistic regression analyses, female (POR, 1.48; 95% CI, 1.17–1.87), Han population (POR, 3.28; 95% CI, 1.67–6.42), and higher altitudes (POR, 2.28; 95% CI, 1.74–3.00) were found to be associated with an increased risk of CHD.

**Table 3:** Analysis of risk factors of CHD in univariate and multivariate logistic regression model

Variable	Univariate model		Multivariate model	
	POR (95% CI)	*P* value	POR (95% CI)	*P* value
Nationality				
Non-Han	Reference		Reference	
Han	2.79 (1.43–5.45)	0.003	3.28 (1.67–6.42)	0.001
Altitude				
≤ 4200 m	Reference		Reference	
> 4200 m	2.22 (1.70–2.92)	< 0.001	2.28 (1.74–3.00)	< 0.001
Gender				
Male	Reference		Reference	
Female	1.47 (1.16–1.86)	0.001	1.48 (1.17–1.87)	0.001

*POR* Prevalence odds ratio, *CI* Confidence interval

## Discussion

This study found that the prevalence of CHD was 6.73 ‰ among children aged 3–19 years in Qinghai province and the prevalence increased with the raising of altitude. The prevalence of CHD was significant higher in female, higher altitude, and Han population.

In this study, the total prevalence of CHD in our study was about 6.73 ‰ among all children, 18 ‰ among Han-race children, and 7 ‰ among Tibetan-race children. The total prevalence of CHD in this study was higher than that from the report by Ma and et al. [4]. Ma and et al. found that the total CHD prevalence during 2006–2008 was about 5.66‰. This difference might be explained by the improvement of diagnosis level in the last decade. The CHD prevalence in Han-race in our study was comparable to studies conducted central and eastern coastal areas of China, which found that the prevalence was 3.7‰-26.6‰ [13]. The CHD prevalence among Tibetan-race children in our study was lower than the results from a previous study in Qinghai Province [14]. Chen and colleague found that the total CHD prevalence in 2006 among Tibetan children was out 7.21‰ [14]. The reason for the decrease in CHD prevalence might due to that the socioeconomic status of Qinghai province and the health condition of local residents have been significantly improved in the past decade. The adoption of a healthy lifestyle in the survey areas and the popularization of knowledge of prenatal and postnatal care have reduced the exposure of pregnant women to CHD risk factors during pregnancy [15].

Our study found that the CHD prevalence in Qinghai province was about 5.86 ‰ and 12.94 ‰ in lower and higher altitude areas, respectively. The results were comparable to findings in Tibet Autonomous Region [8, 9]. Two cross-sectional studies conducted during 2012–2013 found that the total prevalence of CHD varied at different altitudes in Tibet Autonomous Region, with about CHD prevalence of 4.3 ‰-4.6 ‰ in lower altitude (≤ 4200 m) and 12.1 ‰-13.4 ‰ in higher altitude (> 4200 m) [8, 9]. How geographic variation affected the prevalence of CHD in the Qinghai-Tibetan Plateau has not been established. Spontaneous closure of the ductus arteriosus postnatally is impeded by decreased oxygen tension [16], which explains the prevalence of PDA at an altitude < 2500 m and > 4000 m (0.9 ‰ vs 5 ‰, *P* < 0.01). Hypoxemia, when combined with hyperthemoglobinemia and pulmonary arterial hypertension, contribute to the formation of CHD [17].

The top three most common CHD were as follows: ASD (3.5 ‰), PDA (2.1 ‰), and VSD (0.7 ‰). Previous studies on the prevalence of ASD, PDA, and VSD in Tibetan Plateau was 3.3 ‰-3.8 ‰, 1.38 ‰-7.70 ‰, and 0.7 ‰-1.3 ‰, respectively [8, 18, 19]. Compared to previous reports, the CHD prevalence in present study was neither extremely low nor high, as well as the proportion of the main subtypes of CHD (ASD, PDA, and VSD). Compared to male, our study found that female had a higher prevalence of total CHD, ASD, and PDA; the CHD prevalence in both genders increased with raising of altitudes. This was consistent with the results of the Ma’s [4] and Zheng’s reports [8].

Interestingly, the rate of CHD is significantly higher in children under the age of six than in children over that age. At present, we used a two-step method to diagnose CHD. First, a primary screen consisting of a physical examination was conducted on all children, and then conduct cardiac ultrasound diagnosis for the subjects with abnormal physical examination. Nevertheless, many small and medium—sized ASD generally do not have obvious cardiac murmurs and symptoms, so it is difficult to be found without active echocardiographic screening. Of note, ASD was the main type of CHD in older children. This method probably lead to the missed diagnosis of CHD. The results may explain why the prevalence of CHD is significantly higher in children under the age of six than in children over that age.

Interestingly, the Han population had the highest prevalence of CHD when compared to the Hui, Mongol, and Tibetan populations. This is the first report of a significant increase in the CHD prevalence among the Han population residing on the plateau. Although the proportion of Han population was small (18 ‰) in the present study, the difference was statistically significant. The reason for this diversity is unknown. We speculated that the difference can be attributed to altitude sickness between the nationalities. As described by Thomas et al. [20], lived in the highlands (> 2500 m) for ≥ 2000 years, the Aymaras developed several distinctive circulatory adaptations and maladaptations to longtime high-altitude exposure. Among these adaptations, arterial hypoxemia and erythrocytosis are well-known and thought to be an ideal adaptation to life at high-altitudes. Recent studies involving Tibetans, a high-altitude population, had a longer high-altitude resident history, less genetic admixtures, and showed a more advanced high-altitude adaptation pattern of normal hemoglobin level with arterial hypoxemia. These studies demonstrated that Tibetans had advanced high-altitude adaptation when copared to a population living in low altitudes or short time high-altitude residents, which should decrease the prevalence of cardiovascular diseases or defects. Nevertheless, according to some studies, the high-altitude environment adaptation among minority ethnicities could be found in the gene source [21]. However, the population in altitudes ≥ 4000 m in our study were nearly all Tibetans and the prevalence of CHD increased ≥ 50% compared to those lived at 3600 m. The results demonstrated that even with advanced high-altitude adaptation to an altitude > 4000 m, which might have a hostile environment and climate should increased the prevalence of cardiac defects, such as CHD in Tibetans.

This study had several advantages. First, in our study, the multistage random sampling method was used to select counties in Qinghai province; and students in all kindergartens, primary schools, and high schools in selected counties were included and each underwent health examination and CHD screening. The check-up, sponsored by a charity and offered free to the students, was a powerful incentive for parents or their guardians to agree their children to participate the screening. Moreover, the free compulsory education has been successfully implemented in the region, with almost all school-age children receiving primary and secondary education. Therefore, our study population was representative and the results were credible. Second, two-step method was adopted for screening CHD to avoid missed diagnosis. Last but not the least, all clinicians and ultrasound doctors have rich work experience and have been trained many times to ensure the quality of data.

This study had some limitations. First, as a cross-sectional study, the results only reflected the prevalence of CHD in Qinghai province, providing up-to-date data in this geographical region. Long-term follow-up is necessary to determine the temporal trends regarding the prevalence of CHD. Second, the etiological factors related to CHD should be investigated in the future study. Third, this study was performed utilizing the International Classification of Diseases, Ninth Revision codes, and the Clinical Modification code, which bear the potential for inaccuracy of diagnosis, especially when it comes to CHD. It should also be noted that this study may not fully capture or depict all patients with CHD, due to the lack of breadth in ICD codes used.

## Conclusion

The results of study showed that the prevalence of CHD among school-aged children in Qinghai province was 6.73 ‰, and it was higher in female, high altitude area, and Han population.
